# Dataset on effects of nitrogen fertilizer and soil moisture levels on the performance of Water Efficient Maize (WEMA) on Ferric Luvisol and Rhodic Ferralsol soils

**DOI:** 10.1016/j.dib.2023.109543

**Published:** 2023-09-09

**Authors:** Abidemi Ruth. Adebayo, Erick Tshivetsi. Sebetha

**Affiliations:** Food Security and Safety Niche Area Research Group, Faculty of Natural and Agricultural Sciences, North-West University Mafikeng Campus, Private Bag x 2046, Mmabatho 2735, South Africa

**Keywords:** Photosynthetically active radiation, Grain yield, Soil types, WEMA variety

## Abstract

The most important factors affecting maize production are water stress and nitrogen deficiency. A greenhouse experiment was conducted to assess the influence of different N fertilizers and soil moisture levels on the growth and yields of the WEMA variety on two different soils. The experiment was designed in a factorial of 5 × 2 × 2 fitted into a three replicate completely randomized design. Treatments included five N fertilizer rates (0, 60, 120, 180, and 240 kg N/ha), two soil moisture levels [45 and 100% field capacity], and two soil types. The morphological traits, physiological traits, drought indices and agronomic efficiency were determined. The data were analyzed using GenStat, version 11, analysis of variance (ANOVA), and differences in treatment means were assessed with a probability of 5% using the Duncan Multiple Range Test (DMRT). The associations between the measured parameters were examined using regression and correlation analysis. Data were analyzed using analysis of variance (ANOVA) of GenStat, edition 11, and differences in treatment means were tested using the Duncan Multiple Range Test (DMRT) with a probability of 5%. The regression and correlation analyses were used to examine the relationships between the measured parameters.

Specifications Table

**Table:** 

Subject	Agricultural Science
Specific subject area	Agronomy, Soil Science
Type of data	Table Figure
How the data were acquired	Photosynthetically Active Radiation PAR was obtained by using the ACCUPAL Model LP-80 PAR/LAI Ceptometer (Decagon Devices). Plant height: shoot dry matter ratio, plant height: stem diameter ratio, shoot dry matter ratio: plant height: physiological traits, dry matter attributes, ear traits, drought indices, grain yield, yield component and water use efficiency were evaluated as described in this article.
Data format	Raw and Analyzed
Description of data collection	Photosynthetically Active Radiation with the aid of Decagon device, plant height: shoot dry matter ratio, shoot dry matter ratio: plant height as described by Gallegos-Cedillo et al. [6] physiological traits, dry matter attributes as described by Kumar et al. [5,4], ear traits, drought indices, grain yield, yield component and water use efficiency were obtained as described by Abebe and Feyisa [1,2]
Data source location	*· Institution: Research Farm, North –West University* *· City/Town/Region: North –West, Mafikeng* *· Country: South Africa* *· Latitude and longitude;* 25° 48^׳^S, 45°38^׳^E.
Data accessibility	Repository name: Mendeley dataset Data identification number: 10.17632/65bybds4sc.1 Direct URL to data: https://www.mendeley.com/datasets/65bybds4sc/1
Related research article	Nitrogen Fertilizer and Soil Moisture Levels on the Performance of Drought-Tolerant Maize on Ferric Luvisol and Rhodic Ferralsol Soils. Journal of Agriculture and Crops. (2022). 8 (3) 138-151. DOI: 10.32861/jac.83.138.151

## 1 Value of the Data

• The dataset revealed the response of the growth and grain yield of WEMA maize to varying soil types.
• The data indicated the influence of various soil moisture levels on morphological and physiological traits and drought indices of WEMA maize.
• The data presents the influence of different nitrogen rates on dry matter traits, yield component and agronomic and water use efficiencies.
• The dataset revealed the relationship between soil types, soil moisture levels and nitrogen rates on measured parameters.
• A general agronomist and soil physicist can use the data.

## 2 Objective

Water and nitrogen are the most restricting factors for agricultural productivity worldwide, particularly in arid and semiarid countries. In various parts of South Africa, where water and nitrogen are the most limiting nutrient, low yields are particularly prevalent in maize fields. This dataset was collected to assess the respond of Water Efficiency Maize for Africa (WEMA) to different nitrogen fertilizer rates and water stresses on the Ferric Luvisol and Rhodic Ferralsol soils. Therefore, the data can be used to examine the performance of WEMA maize to various nitrogen fertilizer and soil moisture levels on the Ferric Luvisol and Rhodic Ferralsol soils. The data can be used by researchers and farmers as a guideline for identifying the degree of tolerance of WEMA maize to different nitrogen and soil moisture levels on the Ferric Luvisol and Rhodic Ferralsol soils.

## 3 Data Description

The data set revealed the influence of different soil moisture levels and nitrogen fertilizer rates on growth, grain yield and water use efficiency of the WEMA variety on Ferric Luvisol and Rhodic Ferric soil types. The experiment was conducted in a greenhouse using two categories of soil (Ferric Luvisol and Rhodic Ferralsol soil) soil physicochemical as indicated in Table 1. Table 2 expressed the effect of varying N and soil moisture levels on PAR at 6, 8 and 12 weeks after sowing (WAS). Table 3 exhibited the response of plant height: shoot dry weight and shoot dry weight: plant height of cultivated maize plant to soil moisture levels and different nitrogen rates on Ferric Luvisol and Rhodic Ferric soil types at 6, 8 and 12 WAS.

**Table 1:** Physico-chemical properties of the soil types.

Physico-chemical properties	Ferric Luvisol	Rhodic Ferralsol
Sand %	82	85
Silt %	1	1
Clay %	18	14
Texture	Sandy loam	Loamy sand
pH (H_2_O)	4.13	5.60
Total N (%)	0.14	0.22
Phosphorus (mg/kg)	7.00	10.00
Potassium (mg/kg)	235	240
Bulk density (g/cm^3^)	1.80	1.60
Soil water content (g/g)	0.093	0.097
Volumetric water content (g/cm^3^)	0.17	0.16
Soil porosity (%)	68	60
Effective saturation (%)	25	27
Field water-holding capacity (%)	49.5	50.7

**Table 2:** Effect of soil types, soil moisture and nitrogen fertilizer rates on photosynthetically active radiation (nm).

Treatment Factors	6 WAS	8 WAS	12 WAS
**Soil Types**			
Ferric Luvisol	741.50a	318.70b	648.80b
Rhodic Ferralsol	451.20b	537.30a	663.70a
LSD (*p* ≤ 0.05)	28.40	4.70	4.40
**Soil Moisture (%)**			
45	622.90a	76.67a	672.10a
100	569.90b	72.40b	640.40b
LSD (*p* ≤ 0.05)	28.38	0.74	4.41
**N Rates (kg N/ha)**			
0	614.50a	78.00a	611.20d
60	611.20a	76.67b	664.00b
120	565.50b	73.17c	628.80c
180	586.20a	72.88c	716.50a
240	604.40a	71.96d	660.80b
LSD (*p* ≤ 0.05)	44.87	1.18	6.98
**Grand Mean**	596.40	428.00	656.30
**Interaction**			
N rates x soil types	**	**	**
N rates x soil moisture	**	**	**
Soil types x soil moisture	**	**	**
N rates x soil types x soil moisture	**	**	**

**Table 3:** Influence of soil types, soil moisture and nitrogen fertilizer rates on plant height: shoot dry weight ratio and shoot dry weight: plant height ratio.

Treatment Factors	Plant height: shoot dry weight ratio (cm/g)			Shoot dry weight: Plant height ratio (g/cm)		
	6	8	12	6	8	12
**Soil Types**						
Ferric Luvisol	4.55a	1.78a	1.32a	0.30b	0.66b	0.77b
Rhodic Feralsol	3.85b	1.57b	1.25b	0.38a	0.77a	0.89a
LSD (*p* ≤ 0.05)	0.55	0.02	0.02	0.02	0.006	0.06
**Soil Moisture (%)**						
45	3.85b	1.35b	1.23b	0.38a	0.83a	0.88a
100	4.55a	2.01a	1.34a	0.31b	0.61b	0.77b
LSD (*p* ≤ 0.05)	0.55	0.02	0.02	0.02	0.006	0.06
**N Rates (kg N/ha)**						
0	4.52b	1.41d	1.20c	0.30c	0.77b	0.92a
60	6.87a	2.04a	1.34a	0.26d	0.69c	0.84a
120	3.92c	1.89b	1.32a	0.27d	0.57c	0.79b
180	3.34c	1.61c	1.31a	0.40b	0.80a	0.81b
240	2.36d	1.42d	1.26b	0.50a	0.76b	0.77b
LSD (*p* ≤ 0.05)	0.88	0.03	0.03	0.03	0.010	0.09
**Grand Mean**	4.20	1.67	1.29	0.34	0.71	0.83
**Interaction**						
N rates x soil types	**	**	**	**	**	**
N rates x soil moisture	**	**	**	**	**	**
Soil types x soil moisture	**	**	**	**	**	**
N rates x soil types x soil moisture	**	**	ns	ns	**	**

In addition, Table 4 demonstrates the respond of ear characteristics of WEMA maize to different nitrogen fertilizer and soil moisture level under distinct soil types. Table 5 displays the influence of soil types, soil moisture levels, and nitrogen fertilizer rates on dry matter attributes. The response of nutritional quality of grown maize plant as affected by varying nitrogen fertilizer and soil moisture on the Ferric Luvisol and Rhodic Ferric soil types as indicated in Table 6. The influence of soil type, soil moisture and nitrogen fertilizer rates on grain yield and yield components, as represented in Table 7. Table 8 revealed regression between grain yield, physiological traits, dry matter attributes and agronomic efficiency. Furthermore, Tables 9 presents the correlation between yield and ear traits. Similarly, the relationship between the nitrogen fertilizer rates and drought tolerance indices as indicated in Table 10. The data raw of plant height, shoot dry matter ratio, active photosynthetic radiation and ear traits were included in (Excel spread sheets 1 and 2). Likewise, the data raw data of the dry matter attributes, drought indices, nutritional quality, yield and yield components, and agronomic efficiency were comprised of Excel spreadsheets 3 – 7).

**Table 4:** Effect of soil types, soil moisture and nitrogen fertilizer rates on yield attributes of WEMA maize.

Treatment Factors	Ear Height (cm)	Ear Diameter (cm)	Ear Length (cm)	Row Of Kernel/ Ear	Number Of Kernel /Row/Ear	Number Of Kernel/Ear	Kernel Mass (g)	Cob Mass (g)
**Soil Types**								
Ferric Luvisol	99.19b	3.88b	17.22b	11.335b	19.30b	227.30b	66.49b	31.68b
Rhodic Ferralsol	105.10a	4.29a	18.40a	13.30a	26.57a	354.40a	102.35b	39.32a
**LSD (*p* ≤ 0.05)**	1.83	0.20	0.91	0.83	1.23	23.96	1.60	0.82
**Soil Moisture**								
45	92.38b	3.94b	17.28b	11.47b	19.77b	235.00b	70.59b	31.64b
100	111.90a	4.23a	18.34a	13.18a	26.10a	346.70a	98.25a	39.35a
**LSD (*p* ≤ 0.05)**	1.84	0.19	0.90	0.82	1.23	24.00	1.63	0.82
**Nitrogen Rates (kg N/ha)**								
0	85.68d	4.05a	15.39c	12.25b	18.58c	233.10c	56.04d	26.86d
60	95.00c	3.75b	16.38c	11.21b	19.83c	238.30c	71.60c	32.71c
120	108.00b	4.18a	17.84b	13.63a	24.83b	341.40a	88.05b	36.07b
180	108.40b	4.28a	19.93a	12.83a	26.25a	341.20a	102.36a	41.47
240	113.58a	4.20a	19.51a	11.67b	25.17a	300.20b	103.99a	40.39
**LSD (*p* ≤ 0.05)**	2.91	0.30	1.43	1.31	1.95	37.88	2.58	1.30
**Grand Mean**	102.14	4.09	17.81	12.32	22.93	290.90	84.42	35.50
**Interaction**								
N rates x soil types	**	ns	**	ns	ns	**	**	**
N rates x soil moisture	**	ns	ns	ns	ns	ns	**	**
Soil types x soil moisture	**	ns	ns	ns	**	ns	**	ns
N rates x soil types x soil moisture	**	ns	ns	ns	ns	ns	**	**

**Table 5:** Respond of dry matter attributes to soil types, soil moisture and nitrogen fertilizer rates.

Treatment Factors	Translocation of Dry Matter(g)	Post–anthesis Dry Matter Accumulation (g)	Dry Matter Translocation Efficiency (%)
**Soil Types**			
Ferric Luvisol	111.13a	68.10a	46.37a
Rhodic Ferralsol	108.34b	46.70b	38.07b
LSD (*p* ≤ 0.05)	0.93	13.90	0.83
**Soil Moisture (%)**			
45	84.50b	32.10b	36.77b
100	134.97a	82.70a	47.68a
LSD (*p* ≤ 0.05)	0.92	13.90	0.82
**N Rates (kg N/ha)**			
0	119.79a	94.80a	59.70a
60	111.34a	65.00b	44.60a
120	100.84c	41.29c	35.40c
180	106.84b	41.30c	34.80d
240	110.53a	44.40c	36.61c
LSD (*p* ≤ 0.05)	1.47	21.99	1.31
**Grand Mean**	109.73	57.40	42.22
**Interaction**			
N rates x soil types	**	**	**
N rates x soil moisture	**	**	**
Soil types x soil moisture	**	**	**
N rates x soil types x soil moisture	**	ns	**

**Table 6:** Effect of soil types, soil moisture and nitrogen fertilizer rates on the nutritional quality of WEMA maize.

Treatment factors	Starch	Protein	Oil
**Soil Types**			
Ferric Luvisol	59.49a	6.24	3.21a
Rhodic Ferralsol	60.34a	5.70	3.68a
LSD (*p* ≤ 0.05)	1.24	0.48	0.30
**Soil Moisture (%)**			
45	59.53a	6.07	3.36a
100	60.34a	5.87	3.53a
LSD (*p* ≤ 0.05)	1.25	0.48	0.31
**Nitrogen Rates (kg N/ha)**			
0	60.33a	5.74b	3.51a
60	58.41b	5.99a	3.64a
120	61.18a	5.56b	3.45a
180	61.00a	5.85b	3.31a
240	58.66b	6.73a	3.33a
LSD (*p* ≤ 0.05)	1.97	0.77	0.49
**Grand Mean**	59.91	5.97	3.45
**Interaction**			
N rates x soil types	ns	**	ns
N rates x soil moisture	ns	ns	ns
Soil types x soil moisture	ns	**	ns
N rates x soil types x soil moisture	ns	ns	ns

**Table 7:** Yield and yield components of WEMA maize as affected by soil types, soil moisture and different nitrogen fertilizer rates.

Treatment Factor	Yield/pot (g)	Above Biological Yield/Pot (g)	Stover Yield/Pot (g)	Harvest Yield	Water Use Efficiency
**Soil Types**					
Ferric Luvisol	59.10b	197.98b	137.66b	0.29b	0.65b
Rhodic Ferralsol	99.33a	242.84a	141.48a	0.41a	1.15a
LSD (*p* ≤ 0.05)	1.60	2.37	1.72	0.01	0.04
**Soil Moisture (%)**					
45	64.91b	190.30b	124.06b	0.33b	1.10a
100	93.42a	250.51a	155.09a	0.37a	0.70b
LSD (*p* ≤ 0.05)	1.60	2.37	1.72	0.01	0.04
**Nitrogen Rates (kg N/ha)**					
0	49.15e	160.08d	110.93d	0.29e	0.56d
60	64.72d	201.14c	136.42c	0.30d	0.70c
120	88.09c	225.91b	137.88c	0.39b	1.00b
180	104.34a	257.17a	152.83b	0.40a	1.17a
240	97.86b	257.73a	159.88a	0.37c	1.10b
LSD (*p* ≤ 0.05)	2.54	3.75	2.73	0.01	0.07
Grand Mean	80.83	220.41	139.57	0.35	0.90
Interaction					
N rates x soil types	**	**	**	**	**
N rates x soil moisture	**	**	**	**	**
Soil types x soil moisture	**	**	**	**	**
N rates x soil types x soil moisture	**	**	**	**	**

**Table 8:** Regression analysis of physiological indices, agronomic effciency indices and yield of WEMA maize parameters.

Parameters	Equations	R^2^
Tasseling	Y = 0.2879x^2^ – 3.3141x + 81.312	0.95
Silking	Y = 0.4143x^2^ – 5.0317x + 91.04	0.94
Anthesis- Silking Interval	Y = 0.125x^2^ – 1.709x + 9.684	0.88
Translocation	Y = 0.0008x^2^ – 0.2369x + 120.39	0.91
Post- Anthesis location	Y = 0.0018x^2^ – 0.634x + 95.059	0.99
Dry matter Translocation	Y = 0.0008x^2^ – 0.2953x + 59.48	0.99
Yield	Y = -3.6586x^2^– 35.65x + 14.11	0.96
Water use Efficiency	Y = -0.0393x^2^ – 0.3907x + 0.166	0.94
Agronomic Efficiency	Y = -15.16x^2^ – 107.19x +111.44	1

**Table 9:** Correlation relationship between yield, photosynthetically active radiation and ear traits.

Parameters	Yield	Photosynthetically Active Radiation	Ear Height	Ear Length	Ear Diameter	Row Kernel/Ear	Number Of Kernel Row/Ear	Kernel Mass	Cob Mass
Yield	1	0.347⁎⁎	0.651⁎⁎	0.666⁎⁎	0.683⁎⁎	0.570⁎⁎	0.837⁎⁎	0.940⁎⁎	0.883⁎⁎
Photosynthetically Active Radiation	0.347⁎⁎	1	0.048	0.292*	0.232	0.072	0.139	0.363⁎⁎	0.250
Ear Height	0.651⁎⁎	0.048	1	0.489⁎⁎	0.466⁎⁎	0.362⁎⁎	0.726⁎⁎	0.717⁎⁎	0.748⁎⁎
Ear Length	0.666⁎⁎	0.292*	0.489⁎⁎	1	0.457⁎⁎	0.220	0.642⁎⁎	0.658⁎⁎	0.688⁎⁎
Ear Diameter	0.683⁎⁎	0.232	0.466⁎⁎	0.457⁎⁎	1	0.651⁎⁎	0.688⁎⁎	0.642⁎⁎	0.561⁎⁎
Row Kernel/Ear	0.570⁎⁎	.072	0.362⁎⁎	0.220	0.651⁎⁎	1	0.560⁎⁎	0.524⁎⁎	0.355⁎⁎
Number Of Kernel Row/Ear	0.837⁎⁎	.139	0.726⁎⁎	0.642⁎⁎	0.688⁎⁎	0.560⁎⁎	1	0.840⁎⁎	0.814⁎⁎
Kernel Mass	0.940⁎⁎	0.363⁎⁎	0.717⁎⁎	0.658⁎⁎	0.642⁎⁎	0.524⁎⁎	0.840⁎⁎	1	0.901⁎⁎
Cob Mass	0.883⁎⁎	0.250	0.748⁎⁎	0.688⁎⁎	0.561⁎⁎	0.355⁎⁎	0.814⁎⁎	0.901⁎⁎	1

⁎⁎ Correlation is significant at the 0.01 level (2-tailed).

⁎ Correlation is significant at the 0.05 level (2-tailed).

**Table 10:** Correlation relationship between nitrogen fertilizer rates and drought attributes.

Parameters	N rates	DTI	SDI	YI	RDY
N rates	1	-0.274	0.771⁎⁎	0.686⁎⁎	-0.482⁎⁎
DTI	-0.274	1	-.0.008	0.253	-0.521⁎⁎
SDI	0.771⁎⁎	-0.008	1	0.659⁎⁎	0.772⁎⁎
YI	0.686⁎⁎	0.253	0.659⁎⁎	1	-0.592⁎⁎
RDY	0.482⁎⁎	-0.521⁎⁎	-0.772⁎⁎	-0.592⁎⁎	1

⁎⁎ Correlation is significant at the 0.01 level (2-tailed).*Correlation is significant at the 0.05 level (2-tailed). DTI = Drought Tolerance Index, SDI = Sensitivity Drought Index, YI = Yield Index, RDY = Relative Decrease in Yield.

## 4 Experimental Design, Materials and Methods

### 4.1 Description of the Study Area

**Photo 1:**
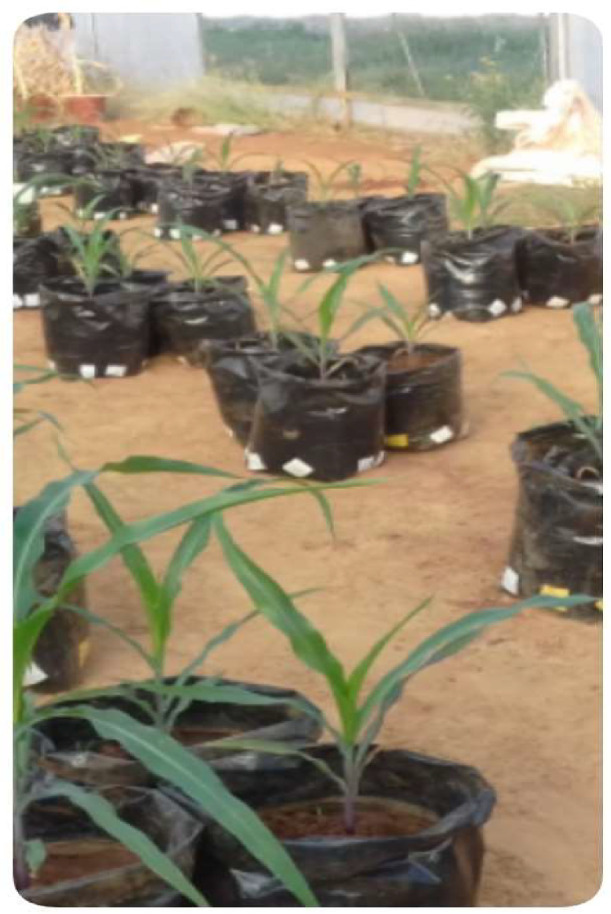
Ferric luvisol soil.

**Photo 2:**
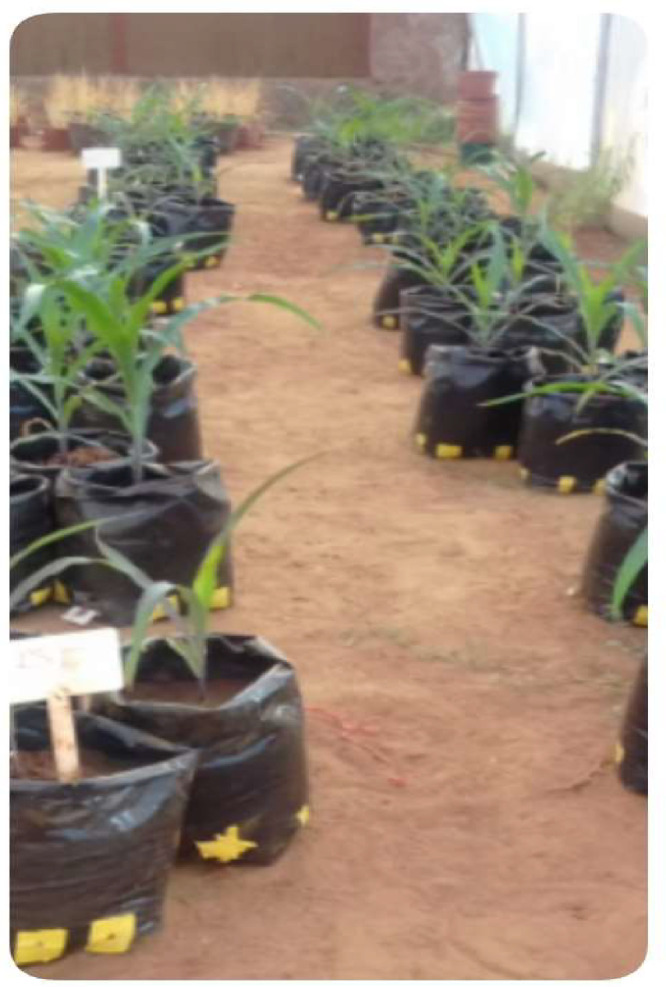
Rhodic ferralsol soil.

**Photo 3:**
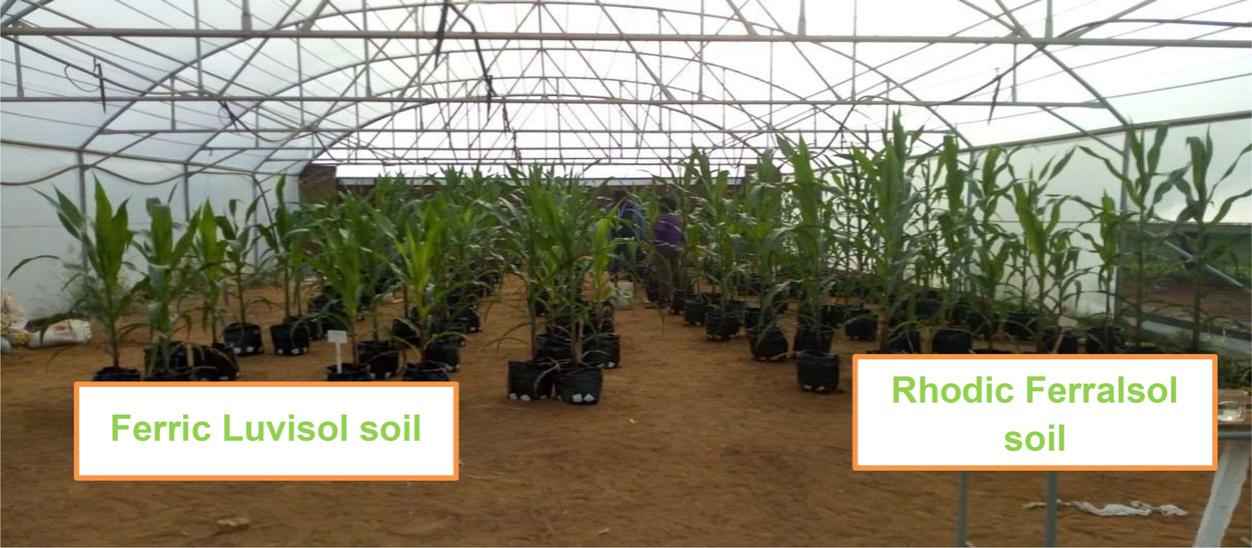
A Green–house structure.

A greenhouse experiment performed at North-West University Research Farm (25° 48^׳^S, 45°38^׳^E). 1012 m above sea level, located in North-West Province. Soil samples were obtained at depths ranging from 0 to 15 cm from from the North-West University's (NWU) Research Farm and the Taung Experimental Station (27° 30^׳^S, 24° 30^׳^E; 1 111 m above sea level) of the Provincial Department of Agriculture. The relative humidity in a greenhouse varies from 63 to 74%, while the temperature is between 24 and 33 °C. In contrast to the loamy sand soil of the Taung experimental site, which is classed as a Rhodic Ferralsol, the soil in Molelwane is a sandy loam and is referred to as a Ferric Luvisol. Soil samples were taken at depths ranging from 0 to 15 cm and analyzed using the standard techniques of the South African Soil Science Guidelines [7] and field capacity for the corresponding fields was determined as described by Kebede et al. [3]. Table 1 displays the results of the preliminary soil analysis. The experiment was set up as a 5 × 2 × 2 factorial with three replications in a completely randomized design block (Photos 1, 2, 3). Five nitrogen fertilization rates (0 (0g/pot), 60 (0.54/pot), 120 (1.08g/pot), 180 (1.62g/pot), and 240 (2.12 g/pot) kg N/ha), two soil moisture levels (45% and 100% field capacity (FC), and two soil types (Ferric Luvisol and Rhodic Ferralsol) were used as treatment factors.

The soil was sieve to remove the plant debris and stone. Plastic pots of 475 mm x 270.70 mm x 339.65 mm dimensions, with perforations sieved using a 6 mm mesh at the bottom, and covered with plumber sellotape to prevent soil loss and leaching, were filled with 18 kg of soil. A total of 180 pots (3 pots/treatment factor) were used. The pots were watered to FC and allowed to equilibrate for eight hours, after which two seeds of WEMA, variety WE3127, were sown in each pot. In order to remove plant debris and stone, the soil was sieved with a 6 mm mesh sieve. Plastic pots (475 mm x 270.70 mm x 339.65 mm) with perforations at the bottom and covered with plumber sellotape to prevent soil loss and leaching were filled with 18 kg of soil. A total of 180 pots (3 pots/treatment component) were used. The pots were watered to field capacity and allowed to equilibrate for eight hours before planting two seeds of WEMA, variety WE3127, in each pot. After 10 days, the seedlings were thinned to one plant per pot. Half of the nitrogen fertilizer (NPK 20:7:3) was applied 10 days after seedling emergence and the other half was applied in the form of lime ammonium nitrate (28% N). The watering treatment was done every two days. The Ferric Luvisol received 1.22 and 2.72 L of water, while the Rhodic Ferralsol received 1.26 and 2.81 L of water, guaranteeing that their respective field capacities (45% and 100% water-holding capacity levels, respectively) could be accommodated. On each soil type and soil moisture level, the total qualities of water applied during the trial period were 130.56 and 58.56 L, respectively, and 134.88 and 61.44 L.

### 4.2 Data Collection

Data were obtained six, eight, and twelve weeks after sowing (WAS). According to Gallego-Cedillo et al. [6], the plant height shoot ratio, shoot dry weight, and photosynthetically active radiation (PAR) data were measured using the ACCUPAL Model LP-80 PAR/LAI Ceptometer, and the dry matter attributes were obtained using the techniques outlined by Kumar et al. [5] and Khatibi et al. [4]. At harvesting, one ear per plant per pot was harvested and shelled. The yield/pot was computed with a moisture content of 12%.

$$\text{Yield}\;=\frac{Dry\;yield\;/pot\;}{100-moisture\;content/100}\left(\text{CIMMYT},\;2013;\;\text{Abebe}\;\text{and}\;\text{Feyisa},\;2017\right)$$

Totalshootbiomass=grainyield+stoveryield(Yada,2011)

$$\text{Harvest}\;\text{Index}\;=\;\frac{Economic\;yield\;\left(g\right)}{Total\;biological\;yield\;\left(g\right)}\left(\text{CIMMYT},\;2013\right)$$

Water use efficiency was calculated as follows:

$$\text{Water}\;\text{use}\;\text{efficiency}\;\left(\%\right)=\frac{Grain\;yield\;g/pot}{Quantity\;of\;water\;applied\left(L\right)}$$

### 4.3 Statistical Analysis

All data obtained were subjected to ANOVA using the GenStat 11th version. The DMRT was used to differentiate differences in treatment means at a 5% probability level. The regression relationship between nitrogen fertilizer rates was assessed using the Excel program, whilst the association between grain yield and PAR was analyzed using the SPSS program. This work confirms the high importance of agricultural sciences in different applications.

## Ethics Statements

This experiment does not involve studies with animals and humans.

## CRediT authorship contribution statement

**Abidemi Ruth. Adebayo:** Conceptualization, Methodology, Data curation, Writing – original draft, Visualization, Investigation, Writing – review & editing. **Erick Tshivetsi. Sebetha:** Data curation, Writing – original draft, Visualization, Investigation, Supervision, Writing – review & editing.

## Data Availability

Dataset on effects of nitrogen fertilizer and soil moisture levels on the performance of Water Efficient Maize (WEMA) on Ferric Luvisol and Rhodic Ferralsol soils (Original data) (Mendeley Data).
